# Nutritional rickets in immigrant and refugee children

**DOI:** 10.1186/s40985-016-0018-3

**Published:** 2016-07-22

**Authors:** Tom D. Thacher, Pawel Pludowski, Nick J. Shaw, M. Zulf Mughal, Craig F. Munns, Wolfgang Högler

**Affiliations:** 1 Department of Family Medicine, Mayo Clinic, 200 First Street SW, Rochester, MN 55905 USA; 2 Department of Biochemistry, Radioimmunology and Experimental Medicine, The Children’s Memorial Health Institute, Warsaw, Poland; 3 Department of Endocrinology & Diabetes, Birmingham Children’s Hospital, Birmingham, UK; 4 Department of Paediatric Endocrinology, Royal Manchester Children’s Hospital, Manchester, UK; 5 The Children’s Hospital at Westmead, Paediatrics and Child Health, Sydney Medical School, University of Sydney, Sydney, Australia; 6 Institute of Metabolism and Systems Research, University of Birmingham, Birmingham, UK

**Keywords:** Vitamin D, Calcium, Nutrition, Metabolic bone, Pediatric

## Abstract

Immigrant and refugee populations bring public health challenges to host nations. In the current global refugee crisis, children are the most vulnerable subpopulation. Diseases that were considered rare in the host nation may be highly prevalent among immigrant children. The prevalence of nutritional rickets is increasing in high-income countries, largely driven by an influx of immigrant populations.

Nutritional rickets is a bone disease in early childhood resulting in bone pain, delayed motor development, and bending of the bones, caused by vitamin D deficiency and/or inadequate dietary calcium intake. The consequences of nutritional rickets include stunted growth, developmental delay, lifelong bone deformities, seizures, cardiomyopathy, and even death.

Nutritional rickets is most commonly seen in children from the Middle East, Africa, and South Asia in high-income countries. Dark skin pigmentation, sun avoidance, covering the skin, and prolonged breast feeding without vitamin D supplementation, are important risk factors for vitamin D deficiency, and combined with a lack of dairy products in the diet, these deficiencies can result in insufficient calcium supply for bone mineralization.

We recommend screening all immigrant and refugee children under 5 years of age from these ethnic groups for nutritional rickets, based on clinical features, and confirming the diagnosis with radiographs of the wrists and knees. Because nutritional rickets is entirely preventable, public health policies must address the need for universal vitamin D supplementation and adequate dietary calcium to protect children from this scourge. Vitamin D supplementation of all infants and children with 400 IU/d during the first year of life and dietary or supplemental intakes of at least 600 IU/d of vitamin D and 500 mg/d of calcium thereafter, will effectively prevent nutritional rickets.

We call on national health authorities of host countries to implement health check lists and prevention programs that include screening for micronutrient deficiencies, in addition to assessing infections and vaccination programs. Due to their high prevalence of vitamin D deficiency, refugee children of all ages from these ethnic groups should be supplemented with vitamin D, beginning upon arrival.

## Background

Caring for immigrant and refugee populations brings with it unique health care needs that pose public health challenges to host nations. Many high-income countries are faced with hundreds of thousands of asylum seekers annually, with increasing numbers in 2014 and 2015, leading to a global refugee crisis. In the midst of this crisis, children are the most medically vulnerable subpopulation [1]. Public health planners need to anticipate and prepare for the medical consequences of an increased influx of refugees and immigrants. This includes planning for an increased frequency of diseases and micronutrient deficiencies that may have a low prevalence in the resident population [2]. One childhood disease that is particularly relevant in immigrants and refugees is nutritional rickets.

**Fig. 1:**
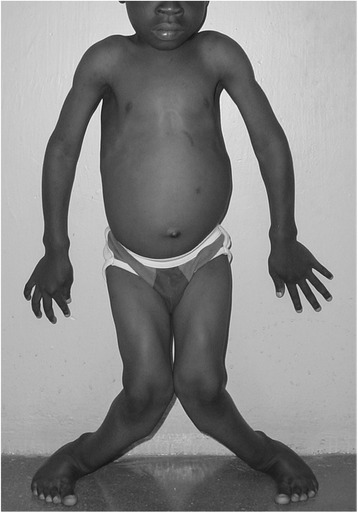
Clinical signs of nutritional rickets. This Nigerian child has severe leg deformities, wrist enlargement, and enlarged costochondral junctions of the ribs

Nutritional rickets is characterized by softening of the growing bones of children, resulting in bone pain, delayed motor development, muscle weakness, and bending of the bones. The bending of bones is most prominent in the legs, manifest as bow leg or knock-knee deformities (Fig. 1). Nutritional rickets results from inadequate vitamin D and/or calcium nutrition, because both nutrients are essential for bones to become mineralized. The clinical consequences of nutritional rickets can include stunted growth, developmental delay, lifelong deformities, pneumonia, hypocalcemic seizures, cardiomyopathy, and even death. Global consensus recommendations for the treatment and prevention of nutritional rickets have been recently published [3, 4].

## Epidemiology of rickets in immigrant children

We participated in the Global Consensus Conference on the Prevention and Management of Nutritional Rickets and conducted a systematic review and grading of the evidence in the English language literature. The search strategy has been described in the recently published consensus recommendations [3, 4]. Recent evidence indicates that the prevalence of nutritional rickets is increasing in high-income countries, with a significant factor being changes in the ethnic composition of the childhood population which is partly related to an increased influx of immigrant populations. Representative incidence and prevalence data are shown in Table 1. The risk of nutritional rickets will likely continue to increase as the proportion of dark-skinned immigrant and refugee children increases in industrialized countries.

**Table 1:** Incidence or prevalence of nutritional rickets in various regions

Country	Population	No. with Rickets	Incidence or Prevalence	Ethnic Groups at Risk
			Incidence (per 100,000/y)	
Australia [5]	National	398	4.9 (age ≤15 y) 2300 (Sudanese)	African (63 %) Refugees (75 %)
United States [7]	Olmsted County, Minnesota	23	24 (age <3y) 220 (black children)	Blacks Somali
Canada [10]	National	104	9–12 (age <3y)	Darker skin Immigrants (24 %)
UK [11]	West Midlands	24	7.5 (age <5y) 38 (South Asian) 95 (Blacks)	South Asian Blacks
UK [13]	England		3.2 (age <15y)	South AsianBlacks
Denmark [19]	National	112	2.9 (age <15 y) 60 (immigrants)	Middle Eastern African
			Prevalence (%)	
Qatar [26]	PHCs	129	23.9 %	
Nigeria [30]	3 PHCs in Jos	8	1.2 % (ages 12–18 months)	
Bangladesh [35]	28 villages	278	1.2 % (ages 1–4 y)	
Turkey [54]	Erzurum Province	39	0.1 % (age <3 y)	

*PHCs* primary health centers

In a study of children with nutritional rickets in Australia, the overall annual incidence in children 15 years of age or less was 4.9 per 100,000. Of the 398 children with rickets, 75 % were refugees, and most were identified as a result of screening in refugee clinics based at general hospitals. Other cases were identified through general hospitals (inpatient or outpatient settings) or non-refugee clinic settings. However, the prevalence in immigrant children was much greater. Nearly all (98 %) of the children with rickets had dark skin pigmentation, and 18 % of girls were partially or completely veiled. Approximately two-thirds were born in Africa [5]. An earlier study found that the increase in the number of cases of rickets in Australia mirrored immigration trends [6].

An increasing incidence of children with rickets has also been documented in the USA. In a population-based study over 40 years in Olmsted County, Minnesota, the incidence of nutritional rickets in children younger than 3 years was 0, 2.2, 3.7, and 24.1 per 100,000 for the decades beginning in 1970, 1980, 1990, and 2000, respectively [7]. Most children with rickets were black. The rising incidence of rickets was temporally associated with an increase in dark-skinned (predominantly Somali) immigrants who have settled in Minnesota since 1994 [8]. The incidence of rickets in black children was estimated as 220 per 100,000. Similarly, a high proportion (71 %) of children with vitamin D deficiency were of Somali origin in Bristol, United Kingdom [9].

In a national survey of pediatricians in Canada, the annual incidence of nutritional rickets was estimated at 9 to 12 cases per 100,000 in children younger than 3 years [10]. Most (89 %) of the children had intermediate or darker skin, but the majority had lived in Canada throughout their lives. Nutritional rickets was associated with breast-feeding in the absence of appropriate vitamin D supplementation.

In the UK, a survey of pediatricians identified 24 cases of symptomatic vitamin D deficiency in children less than 5 years of age. The overall annual incidence was 7.5 per 100,000, but children of South Asian ethnic origin had a five-fold greater incidence of 38 per 100,000, and those of black African or African-Caribbean ethnic origin had an incidence of 95 per 100,000 [11]. In a group of 124 South Asian children, 6–36 months of age, in Manchester, UK, 1.6 % had evidence of nutritional rickets confirmed by x-rays [12]. An analysis of historical trends of hospital admissions for rickets in England from 1963 to 2011 found that hospitalization rates for rickets are now the highest in five decades [13]. South Asian (33 %) and blacks (33 %) were disproportionately overrepresented among children with rickets. Changes in the population structure with a larger proportion of children from ethnic minorities likely accounts for a large part of the increased incidence. Between 2001 and 2009, the number of white children in England declined 6 % and the number of non-white children increased by 19 % [13]. The incidence of hypocalcemic seizures, another manifestation of severe vitamin D deficiency, was greater in children of South Asian or Black ethnicity compared with children from white ethnic backgrounds in the UK [14]. Data from Glasgow, Scotland document a quadrupling of symptomatic vitamin D deficiency (mostly rickets) between 2002 and 2008, with the greatest increase in patients with an ethnic background of the Sub-Saharan region of Africa, North Africa or the Middle East [15].

Dark skinned races consistently have a higher risk of rickets. The greater the number of people at risk, the greater the number of children with nutritional rickets. Exemplary national statistical data from UK [16], Germany [17], and Australia [18] show increasing population percentages of current or former immigrants from regions where people are ethnically dark-skinned and/or traditional diets are low in calcium. The proportion of the white population in England/Wales has decreased from 94.1 % in 1991 to 86 % in 2011, replaced mainly by an increase in people originating from countries with ethnically dark skin, which is a result of both higher birth rates and immigration [16]. In Germany, for example, between 2008 and 2015, the number of immigrants from Asia has increased 1.9 fold and from Africa 1.6 fold, compared with an increase of 1.3 fold in immigrants from other European countries [17]. Between 1996 and 2013, Australia’s overseas-born population grew by 51.2% to 6.4 million people, now comprising 28 % of Australia’s population [18]. The greatest increases were observed in immigrants from India (4-fold) and China (3-fold). On the other hand, we acknowledge that already resident risk groups (former immigrants, second generation) also tend to increase in numbers more rapidly in many countries than the “historically” white population.

Among 112 patients with nutritional rickets in Denmark, 74 % were immigrants. The overall incidence of nutritional rickets in children aged under 15 years was 2.9 per 100,000, but among immigrant children born in Denmark, the incidence was 20-fold greater at 60 per 100,000 [19]. The incidence of nutritional rickets declined in ethnic Danish children from 5.0 to 2.0 per 100,000 per year (age <3 y) between 1985 and 1994 and 1995–2005. During the same interval, the overall incidence of nutritional rickets increased from 1.7 to 2.9 per 100,000 per year (age <15y), respectively. Among immigrant girls older than 4 years of age, 78 % were veiled [20]. A report from Spain described nutritional rickets in three teenagers of Pakistani origin [21]. Nutritional rickets has been reported in infants immigrating to Israel from Ethiopia [22].

Immigrant and refugee children typically come from countries with a high incidence of nutritional rickets. Many of these countries are in the tropics with abundant sunshine. The highest prevalence of nutritional rickets is found in children in the Middle East, Africa, and South Asia, corresponding to the sites of origin for many immigrants (Table 1). Vitamin D deficiency is highly prevalent in the Middle East and North Africa, in part related to covering by clothing [23]. For example, reports from Yemen [24], Jordan [25], Qatar [26], Saudi Arabia [27, 28], and Turkey [29] all indicate that nutritional rickets is an important condition in children from the Middle East. Nutritional rickets is prevalent in sub-Saharan African countries, like Nigeria [30], Ethiopia [31], and The Gambia [32], and in South Asian countries, like India [33] and Bangladesh [34, 35]. However, nutritional rickets in children is not limited to these countries [36].

The burden of vitamin D and dietary calcium deficiency is not limited to children. The lack of mineral supply to the growth plate in children creates rickets and bone deformities, whereas the same lack of mineral supply in adults causes osteomalacia in mature bone. Both rickets and osteomalacia are associated with muscle weakness and hypocalcemic complications. While the diagnosis of rickets is easily made, diagnostic criteria for osteomalacia in adults are not well established, and there is a huge dark figure of unrecognized osteomalacia in adults. A post-mortem study in Northern Europe suggested that 25 % of the population is affected by osteomalacia [37].

## Vitamin D and calcium

Vitamin D is produced in the skin by ultraviolet radiation from sunlight, and this is the principal source of vitamin D for most populations. Dark skin pigmentation reduces the quantity of vitamin D produced for a given amount of solar ultraviolet radiation, predisposing children with dark skin to vitamin D deficiency. Compared with the sunny tropical countries of origin of many immigrants and refugees, most high income countries are at higher latitudes and only receive sufficient ultraviolet light during summer months. Colder climates lead to greater covering of the skin with clothing, which prevents production of vitamin D in covered skin. Relocating to high latitude countries from sunny tropical regions puts high risk children at even greater risk of nutritional rickets. Australia is an exception to this, having a temperate and tropical climate. Here too, nutritional rickets is seen amongst the refugee population [5].

Very few foods are naturally rich in vitamin D, so in the absence of adequate sun exposure, children must rely on oral intake of vitamin D- fortified foods or vitamin D supplements to prevent vitamin D deficiency. Nutritional rickets can effectively be prevented by ensuring a vitamin D intake of at least 400 IU/d during the first year of life and 600 IU/d thereafter. However, immigrant and refugee children may not consume commonly fortified staple foods, due to dietary preferences, or they may migrate to countries that do not practice food fortification.

**Fig. 2:**
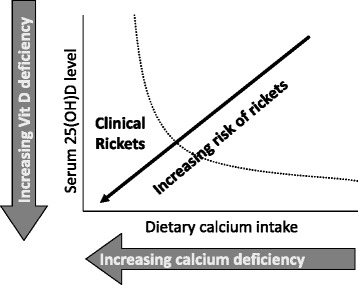
Vitamin D and calcium interaction. The risk of nutritional rickets is a function of both calcium intake and vitamin D status. Clinical rickets develops when the threshold is crossed where bone mineralization is impaired in growing bones

The risk of nutritional rickets is a function of both vitamin D status and calcium intake. Although rickets can result from vitamin D deficiency or calcium deficiency, more commonly these two conditions interact to increase the risk of developing rickets (Fig. 2). The combination of low vitamin D status and inadequate calcium intakes poses a very high risk for rickets in growing children and osteomalacia when growth has ceased. If their diet does not include milk and dairy products, individuals will not likely meet their dietary calcium requirements. Children with calcium intakes below 300 mg/d are at high risk of nutritional rickets from calcium deficiency [33, 38, 39].

Vitamin D status is measured by the serum 25-hydroxyvitamin D [25(OH)D] concentration, which has a half-life of 15 days [40]. Optimal 25(OH)D concentrations are >50 nmol/L (>20 ng/mL). Low vitamin D status in immigrants can result from reduced sun exposure during the winter, modest clothing for cultural and religious reasons, reduced cutaneous vitamin D synthesis due to dark skin, a low intake of vitamin D- fortified foods, and infrequent use of vitamin D supplements [41]. Because the concentration of vitamin D in breast milk is low, prolonged breast feeding without vitamin D supplementation increases the risk of vitamin D deficiency and rickets, which has been described in immigrant children [42]. In Norway, 92 % of people of Pakistani ethnicity had 25(OH)D values below 50 nmol/L [41], and the majority of recently settled immigrant groups from the Middle East, South Asia and Africa had 25(OH)D values below 50 nmol/L [43]. Vitamin D deficiency was also common among Pakistani immigrant children in Denmark, where no food fortification with vitamin D is mandated [44]. Among children with a diagnosis of vitamin D deficiency in Bristol, UK, 71 % were of Somali origin [9]. Vitamin D deficiency was the most common reason for referral of children, mostly from Africa, to a refugee health clinic in Australia, accounting for 39 % of referrals [45].

Women who are vitamin D deficient during pregnancy give birth to infants who are vitamin D deficient and at risk for nutritional rickets and hypocalcemic seizures early in infancy [46]. Somali immigrant women were reported to have a high prevalence of inadequate vitamin D status (25(OH)D < 50 nmol/L) with rates of 90 % in Finland and Norway [47, 48], and the majority of their infants also had low vitamin D status. A recent meta-analysis of dark-skinned migrant populations showed that immigrants from the extended Middle East and sub-Saharan Africa had a high prevalence of vitamin D deficiency (65 and 56 %, respectively) [49]. The authors recommended that migrants at high risk be educated, screened, and monitored for vitamin D deficiency. Severe vitamin D deficiency (25(OH)D <25 nmol/L; <10 ng/mL) was noted in 38 % of Italian and in 76 % of migrant newborns and in 18 % of Italian and 48 % of migrant mothers [50]. A linear decrease of 25OHD levels was found with increasing skin pigmentation. The authors emphasized that a prevention program with vitamin D supplementation should be urgently considered.

## Public health action

Due to the potential of significant morbidity and mortality from untreated nutritional rickets, we recommend screening immigrant and refugee children under 5 years of age from the Middle East, Africa and South Asia, for nutritional rickets, based on clinical features. Nutritional rickets should be suspected in children with a low height for age, delayed walking, leg pain with walking, enlarged wrists or costochondral junctions, or bowing of the long bones in the legs [51]. These signs and symptoms can be assessed with a clinical examination at the initial health assessment of immigrant and refugee children. Confirmation of active rickets requires plain radiographs of the wrists and knees, which show characteristic features in the growth plates, consistent with inadequate mineralization of growing bone [52]. Elevated serum concentrations of alkaline phosphatase and parathyroid hormone, and low concentrations of phosphorus and 25(OH)D are consistent with nutritional rickets. Treatment of nutritional rickets involves provision of treatment doses of calcium and vitamin D, with monitoring of biochemical and radiologic response.

Nutritional rickets is an entirely preventable disease. Primary prevention of nutritional rickets in immigrant children from dark-skinned ethnic risk groups should be based on vitamin D supplementation of all infants and children with 400 IU/d during the first year of life and 600 IU/d thereafter. Supplementation is the quickest way to correct vitamin D deficiency and can effectively prevent rickets in children. Because of the resurgence of nutritional rickets in a predominantly ethnic minority population in Birmingham, UK, the Healthy Start vitamin D supplementation program was universally implemented for pregnant and lactating women and young children. Key factors included a public awareness campaign about the importance of vitamin D, the widespread availability of the supplements in the community and their introduction at age two weeks. As a result, the incidence of symptomatic vitamin D deficiency fell from 120 to 49 per 100,000 children under 5 years, despite only 17 % adhering to supplement use [53]. Far more effective was an infant vitamin D supplementation program in Turkey, which nearly eradicated nutritional rickets [54, 55]. The most significant step was that the Ministry of Health distributed vitamin D supplements to every newborn throughout infancy at no financial cost to families through its network of primary care units and maternal–child health centers. Any intervention for children requires educational and behavioral training of parents, and further research is needed to identify the most effective educational programs.

Primary prevention of rickets must also ensure adequate dietary calcium intake of at least 500 mg/d. This is typically achieved by provision of sufficient milk intake. During infancy, the infant’s calcium requirements can be met by exclusive breast feeding or formula. Whole milk can be introduced after the first year of life, and a 250 ml serving of cow’s milk contains approximately 300 mg of calcium.

It is noteworthy that micronutrient deficiencies such as vitamin D rarely occur in isolation. Refugee children are also often found to be deficient in iron, folic acid, zinc, vitamin A, amongst others. Therefore, preventive measures should include screening for such dietary deficiencies. In view of the current refugee crisis, the programmatic implementation of refugee health assessments will vary, based on individual host nation’s public health infrastructure and societal values, in addition to other factors. We believe that refugees from countries with a high prevalence of dangerous infectious diseases (e.g., tuberculosis), micronutrient deficiencies, or suboptimal vaccination programs, should have obligatory health screening, either at the point of entry, or at least at the place of their new temporary domicile. Appropriate information leaflets in several languages should be produced to inform refugees about the basis for mandatory health screening and the national public health care program. These mandatory measures should not be negotiable; otherwise they pose a health risk to themselves or the host population. Such a screening service and integration into prevention programs will be very costly, labor intensive and require electronic registration systems that allow tracking individuals for legal and health reasons, a common standard in many European countries.

## Conclusion

Nutritional rickets is common in immigrant and refugee children from the Middle East, Africa, and South Asia. Dark skin pigmentation, sun avoidance, covering the skin, and low dietary calcium intake are important risk factors. The escalating number of immigrant and refugee children is a factor in the increased prevalence of rickets in high income countries. Public health policies must address the need for vitamin D supplementation and adequate dietary calcium to protect children from this fully preventable scourge. We call on national health authorities of host countries to implement health check lists and prevention programs that include screening for micronutrient deficiencies, in addition to assessing infections and vaccination programs. Due to their high prevalence of vitamin D deficiency, refugee children of all ages from these ethnic groups should be supplemented with vitamin D, beginning upon arrival.

## Abbreviations

25(OH)D: 25-hydroxyvitamin D
IU: International units
PHCs: Primary health centers
